# Different mutation patterns of *Plasmodium falciparum *among patients in Jimma University Hospital, Ethiopia

**DOI:** 10.1186/1475-2875-9-226

**Published:** 2010-08-07

**Authors:** Teferi Eshetu, Nicole Berens-Riha, Sintayehu Fekadu, Zelalem Tadesse, Robert Gürkov, Michael Hölscher, Thomas Löscher, Isabel Barreto Miranda

**Affiliations:** 1 Department of Infectious Diseases and Tropical Medicine, Ludwig Maximilians University, Leopoldstrasse 5, 80802 München, Germany; 2 Department of Microbiology, Parasitology and Immunology, Jimma University, Jimma, Ethiopia; 3 Department of Paediatrics, Jimma University, Jimma, Ethiopia; 4 Department of Internal Medicine, Jimma University, Jimma, Ethiopia; 5 Department of Otorhinolaryngology, Head and Neck Surgery, Ludwig Maximilians University, Munich, Germany

## Abstract

**Background:**

The emergence of drug resistance is a major problem in malaria control. Combination of molecular genotyping and characterization of mutations or single nucleotide polymorphisms (SNPs) correlated with drug resistance can provide information for subsequent surveillance of existing and developing drug resistance patterns. The introduction of artemether/lumefantrine (AL) as first-line treatment, never used before in Ethiopia, allowed the collection of baseline data of molecular polymorphisms before a selection due to AL could occur.

**Method:**

97 patients with uncomplicated falciparum malaria were recruited from April to June 2006 and treated with either AL, quinine (Q) or atovaquone/proguanil (AP) in Jimma University Hospital, Ethiopia. Mutations or SNPs associated with resistance to these drugs were analysed by RFLP (*pfdhfr*, *pfmdr1*) and sequencing of the target genes (*pfcytb*, *pfserca *).

**Results:**

SNPs previously reported to be associated with resistance to the study drugs were identified in recrudescent and treatment sensitive isolates. A total of seven recrudescences were obtained. The *pfmdr1 *N86Y mutation was found in 84.5% of isolates. The triple mutation 51I,59R,108N of the *pfdhfr *gene occured in high frequency (83.3%) but no *pfcytb *mutation was detected. Sequencing showed a variety of previously described and new mutations in the *pfserca *gene.

**Conclusion:**

The prevalence of mutations was in accordance with the expected patterns considering recent drug regimens. The broad introduction of AL and the cessation of former drug regimens might probably change the current distribution of polymorphisms, possibly leading to decreased sensitivity to AL in future. Continuous surveillance of molecular patterns in this region is, therefore, recommended.

## Background

Malaria is still one of the leading health problems in our time. Most cases and deaths occur in Sub-Saharan Africa. Malaria is endemic in large parts of Ethiopia including the town of Jimma and its surroundings with most cases occurring from September to December and April to June during and after the rainy seasons [1]. High levels of drug resistance of *Plasmodium falciparum *strains against anti-malarials, first chloroquine and later sulphadoxine/pyrimethamine (SP), resulted in new drug policies in Ethiopia. In 2004, the first-line treatment recommendation was switched from SP to artemether-lumefantrine (AL), an artemisinin-based combination therapy (ACT) [1-3]. Due to a shortage in supply, Quinine was used for several months instead. AL was introduced by the underlying study in this region. ACT combines the potential of rapid reduction of the parasite burden and elimination of remaining parasites due to longer-acting partner drugs [4]. The correlation between distinct SNPs and anti-malarial drug resistance or clinical outcome has been widely discussed. Mutations in the *Plasmodium falciparum *multi-drug resistance (*pfmdr1*) gene have been associated with resistance to chloroquine, quinine, mefloquine, lumefantrine and artemisinin [5-8]. Increased sensitivity to (Dihydro-)Artemisinin in the presence of wild type codon 86 in *pfmdr1 *and *in vivo *selection of *pfmdr *86N during AL treatment has been reported [9-12].

The target structure for artemisinins was first described by Eckstein *et al *in 2003, abandoning other theories about the mode of action. PfATP6, a SERCA-type ATPase (*pfserca*) of *P. falciparum*, is inhibited by artemisinins [13]. An L263E replacement and other mutations decreased sensitivity to artemisinins [14]. Since then, further resistance-related mutations concerning artemisinins have been detected in laboratory strains and field isolates associated with *in vitro *resistance [15-17]. Recent reports about high *in vivo *tolerance of artemisinin-based combination therapy (ACT) and artesunate monotherapy in Cambodia and Thailand are all the more alarming [18-20].

In this study, polymorphisms related to drug resistance were investigated. Mutations of the genes *pfmdr1 *(codon 86) and *pfdhfr *(codon 16, 51, 59, 108, 164), the latter associated with proguanile resistance, were analyzed by RFLP. Regions in the *pfserca *and *Plasmodium falciparum cytochrome B (pfcytb) *gene related to artemether and atovaquone resistance, respectively, were sequenced.

## Methods

### Study area and population

The study was performed at the Jimma University (JU) Hospital in the city of Jimma, 1,700 m above sea level and 335 km south west of Addis Ababa, Ethiopia. Patients over five years of age (mean 19.1, range 6-50) with parasitologically proven uncomplicated falciparum malaria were recruited from April until June 2006. Written consent was obtained from either the patient or a parent/legal guardian. The study was conducted concomitantly with the previous published work on ototoxicity of artemether/lumefantrine in comparison with quinine and atovaquone/proguanil and was approved by the Jimma University Ethical Committee and is registered with Clinical Trial.gov, Number NCT00451139 [21].

### Procedures

Blood samples were obtained at days 0, 7 and 28 as well as on any day until day 90 in case of re-occurrence of symptoms suggesting malaria [22]. Aliquots of 10 μl of capillary blood were spotted to Whatman 3 MM Chr filter paper, air dried, and stored at ambient temperature for later molecular analysis. The definition of the treatment outcome followed the WHO draft protocol for areas with low or moderate malaria transmission [2].

Parasite DNA was extracted from blood spots on the filter paper by the Chelex method [23]. The species was identified by nested polymerase chain reaction (PCR) [24]. Single and multiple clone infections from day 0 were determined by amplification of *msp1 *and *msp2 *genes. To distinguish between re-infection and recrudescence, genotyping by amplification of *msp1*, *msp2 *and enzymatic digestion (RFLP) of the PCR products was performed between the pair of samples [25].

To amplify the *pfmdr1 *and *pfdhfr *gene, a nested PCR was used, the polymorphisms were detected by RFLP [26,27]. The PCR products were separated in 2% agarose gels stained with ethidium bromide and visualized under UV light. The regions of interest of the *pfcytb *and the *pfserca *gene were amplified by established PCR procedures [28,29]. The amplified gene fragments were purified from gel using Ultrafree-DNA extraction kit. Cycle sequencing was done with the BigDye Terminator Cycle Sequencing Kit and products analysed on the ABI3730 sequencer. Primers used for sequencing were those from the PCR. Sequences were verified using templates from two independent amplifications of the same DNA sample. Sequencing analysis was performed from both directions for each template. Sequences were analysed using the programme Bioedit and the NCBI blast function for comparison with sequences published in the GenBank database. (Reference strain for *pfserca*: Dd2, accession number: AB121053 and 3D7, acc no.: AL844501.1; for *pfcytb*: 3D7, acc. No.: AF069605)

## Results

97 patients were included in the study with 30 patients receiving AL, 35 Q, and 32 AP. Clinical and parasitological efficacy as well as baseline data were described elsewhere in detail [21]. No treatment failure occured before or on day 7 in any treatment group. Until day 28, three patients in the Q group and two in the AP group presented with PCR-confirmed recrudescent falciparum malaria. Later, one recrudescence was detected in a patient in the Q group on day 40, and possibly another on day 70 in the AL group (Table 1).

**Table 1:** Clinical and parasitological treatment failures

PCR-corrected failure rates*	AL	Q	AP
Number of patients with recrudescence/total number of patients (day)	1/30 (70)**	4/35 (24, 28, 28, 40)	2/32(28, 28)
Drug resistance associated polymorphisms	1. *pfserca *gene 2. *pfmdr *codon 86N	1. *pfmdr *codon 86N	1. *cytb *gene codon 268 2. *dhfr *triple mutation (51I+59R+108N) 3. *dhfr *codon A16V
Polymorphisms in recrudescent samples n (%)	1. 0 (0) 2. 0 (0)	1. 0 (0)	1. 0 (0) 2. 2 (100) 3. 1 (50)

*P. falciparum: Genotyping by PCR and RFLP patterns of the *msp-1 *and *msp-2 *gene

**Possible Recrudescence

The overall prevalence of *pfmdr1 *mutations was high (84.5%) (Table 2). In the Q group, all four treatment failures showed the *pfmdr1 *mutation at codon 86Y, but also 90.0% of the clinical sensitive samples presented with the mutation. In the AL group, four samples showed the wild type, one mixed (wild type and mutation); the remaining 25 samples exhibited only the mutation.

**Table 2:** Prevalence of *pfdhfr *and *pfmdr1 *mutations.

Genetic Polymorphism	Overall prevalence rate mutation/total n (%)
DHFR A16V	1/97 (1.1)
DHFR N51I	83/84* (98.8)
DHFR C59R	85/97 (87.6)
DHFR S108N	97/97 (100.0)
DHFR S108T	0/97 (0.0)
DHFR (51I,59R,108N)	70/84 (83.3)
DHFR I164L	0/97 (0.0)
pfmdr N86Y	82/97 (84.5)

*RFLP outcome not distinguishable for 13 samples

The expected high prevalence of the polymorphisms at codons 51I, 59R, and 108N of the *pfdhfr *gene was found. Both recrudescent strains in the AP group showed the triple mutations (51I, 59R, 108N). The only A16V mutation in the AP group was found in the clinical symptomatic treatment failure (Table 1).

The *pfserca *gene was amplified and sequenced from the codons 230 to 463 and 600 to 790. Sequencing was intended for all 30 AL samples including the recrudescent strain, amplification failed due to long storage conditions of the DNA and general problems with these particular PCRs for the day 0 counterpart of the one possible recrudescent sample on day 70 and for many PCRs with the ATP1 and ATP2 primers. All gained sequences could be identified as part of the *pfserca *gene with the reference strain Dd2. 12 mutations or SNPs were identified, six new and six described. The previously reported E431K mutant codon was the most frequent occurring in seven isolates [29,30]. Each of the other genotypes was detected only once. No sample showed more than two mutations. Three of the new mutations were non synonymous and the other three synonymous (Tables 3 and 4). The sample from day 70 yielded no mutation in the *pfserca *gene.

**Table 3:** Prevalence of wild type and mutant codons in the *pfserca *gene

Codon	Wild type n (%)	Mutation n (%)
E237A	6/7 (85.7)	1/7 (14.3)
H243Y	7/7 (100.0)	0/7 (0.0)
L263E	7/7 (100.0)	0/7 (0.0)
L263L	6/7 (85.7)	1/7 (14.3)
L402V	14/15 (93.3)	1/15 (6.7)
E431K	5/12 (41.7)	7/12 (58.3)
N460N	12/12 (100.0)	0/12 (0.0)
A623E	23/24 (95.8)	1/24 (4.2)
A630S	23/23 (100.0)	0/23 (0.0)
R682R	22/23 (95.7)	1/23 (4.4)
N683E	22/23 (95.7)	1/23 (4.4)
N683K	22/23 (95.7)	1/23 (4.4)
K766K	26/27 (96.3)	1/27 (3.7)
K767E	26/27 (96.3)	1/27 (3.7)
K767R	26/27 (96.3)	1/27 (3.7)
S769N	26/27 (96.3)	127 (3.7)
K771E	25/25 (100.0)	0/25 (0.0)
K776N	24/24 (100.0)	0/24 (0.0)

Fragments spanning codons 230 to 463 and 600 to 790, respectively, ref. [29])

**Table 4:** List of *pfserca *non synonymous and synonymous mutations found.

Nucleotide mutation	amino acid (NS; *Syn*)	No. of mutations	Reference
A709C	**E237A**	1	New
C726T	**H243Y**	0	29
T787G, T788A	**L263E**	0	29
A789G	*L263L*	1	New
1204G	**L402V**	1	29
G1291A	**E431K**	7	29
T1380C	*N460N*	0	30
C1867A	**A623E**	1	29
G1887T	**A630S**	0	29
A2045G	*R682R*	1	New
A2046G, T2048A	**N683E**	1	29
T2048A	**N683K**	1	30
A2297G	*K766K*	1	New
A2298G	**K767E**	1	New
A2299G	**K767R**	1	New
G2305A	**S769N**	1	29
A2310G	**K771E**	0	29
G2327T	**K776N**	0	29

Fragments spanning codons 230 to 463 and 600 to 790, respectively, ref. [29])

No mutations were detected in the amplified regions of the *pfcytb *gene of 2 recrudescent strains in the AP group.

## Discussion

High cure rates of AL have also been reported from other recent studies with 28 days of follow-up in Ethiopia [31,32]. For the detection of late recrudescences, follow-up periods longer than 28 days seem more appropriate. In this study, genotyping indicated a recrudescence on day 70 in one patient treated with AL. The msp-1 gene and especially the RFLP results with different enzymes (Hinf III, Dde I, Rsa I) of the msp-2 gene showed the same molecular pattern for the two samples from day 0 and day 70 [25]. Additionally, a short sequence of the *msp-1 *gene was amplified, as the amplification of the *pfserca *sequence failed. The two sequences fully matched, blasting showed a difference between the Ethiopian sequence and other published strains from different regions of 90-96% [33]. Though, re-infection with a very similar clone not to be differentiated by molecular methods is still possible regarding the moderate transmission area. This particular molecular RFLP pattern was found in almost a quarter of all samples. Overall, at least six different patterns in different combinations, four being predominant, were observed. (Data not shown) The two particular samples from day 0 and day 70 showed the tyrosine mutation at codon 86 of the *pfmdr1 *gene.

The previously described mutant codon S769N being associated with *in vitro *resistance in French Guiana was found in a clinical sensitive sample [15,17]. Other recently published mutations were not detected in the amplified regions of the study samples but the *pfserca* gene was only partly sequenced for 31 samples [34]. Overall, 12 different mutations were observed in an AL-naive population, some of these mutations seem to be globally distributed as reports from Asia and South Africa indicate and not to be associated with drug resistance or caused by drug pressure [34]. The next step will be the comparison of the polymorphisms in these isolates with about 350 *P. falciparum *samples, recently collected in Jimma area. Almost four years after the broad introduction of AL in Ethiopia, selection or disappearence of certain mutations may have occured.

A study conducted in southern Ethiopia prior to the introduction of ACTs showed a prevalence of *pfmdr1 *86Y of 81% and of the mutant *pfcrt *76 of 100% [35]. High prevalence rates of the *pfmdr1 *mutation 86Y were therefore expected. Q has been used for decades in Ethiopia as second line treatment and treatment for severe malaria, treatment failures occured sporadically. Exact data were not published. Due to intensive use in Thailand for example, failure rates with Quinine raised dramatically. Attempts to show a clear correlation of drug resistance with molecular patterns has failed so far, mutations in the *pfmdr1 *gene were discussed [36]. All treatment failures in the Q group showed the SNP of *pfmdr *86Y associated with chloroquine resistance but increased sensitivity to quinine in *in vitro *studies. No selection to 86N in the recrudescent samples was observed but the small sample size and few treatment failures allow no conclusions [37].

The high prevalence of *dhfr *mutations is likely caused by wide use of proguanil, active metabolite of sulphadoxine/pyrimethamine, prior to AL introduction in the area. In the above cited study from Ethiopia, the *pfdhfr *mutations N108, I51 and R59 were present in 100%, 97% and 90%, respectively, of all investigated samples, the *pfdhfr *triple mutations (51I+59R+108N) occurred in 87% of the isolates [35]. Another study from Jimma reported 100% prevalence of the 108N and 51I mutations, and 54% prevalence of the *pfdhfr *triple mutation [38]. The occurrence of the triple mutations in both recrudescent isolates of the AP group was therefore very likely. Nevertheless, the idea of these mutations being a necessary but not sufficient cause of resistance to proguanil is supported. The circumstance that the only mutation at codon A16V occurred in the recrudescent sample may have contributed at least to the late treatment failure at day 28.

Although the combination AP has never been broadly used in the study region before, it simply served as negative control in the ototoxicity trial, two parasitological failures occurred, one was clinical symptomatic. Treatment failures have been reported from Africa. Some have been associated with mutations in the *cytb *gene, since *in vitro *resistance to AP was correlated especially with mutations at codon 268 of that gene [39-41]. A molecular survey from Ethiopia and Gabon detected no mutations in the *pfcytb *gene of samples from Ethiopia but several different mutations in 10% of Gabonian samples, although AP was not in use in both regions [42]. Spontaneous mutations are rare but seem to occure independent from drug pressure. Both recrudescent isolates in this study showed no mutations in the amplified sequence containing codon 268 but not the whole gene was sequenced and other mutations are possible. Treatment failures may also be due to limited bioavailability in some patients. However, as AP is highly recommended as prophylaxis for travellers to Ethiopia, a failure rate of 6.3% was quite alarming.

## Conclusion

As expected, there were no signs of clinical or parasitological failures in the AL group except for one possible very late recrudescence on day 70 at the time of ACT introduction. The patterns of mutations in general fit with the situation of long-lasting chloroquine and SP usage before the presence of ACT in this area. Q is the national second-line treatment and backup, however its use will be compromised by the degree of resistance shown in our results. AL seems to be the best treatment option and must be available consistently. Moreover continuous surveillance should be established in the area for AL as data from South East Asia showed decreased susceptibility of *P. falciparum *for ACT several years after introduction. A similar development could possibly be expected in Ethiopia.

## Competing interests

The authors declare that they have no competing interests.

## Authors' contributions

TE carried out the clinical study, participated in the molecular genetic studies and helped to draft the manuscript. NBR carried out the molecular genetic studies and the sequence alignment and drafted the manuscript. SF and ZT participated in the clinical studies. RG and MH participated in the design and coordination of the clinical study. TL was PI of the study, participated in its design and coordination. IBM coordinated and designed the study and helped to draft the manuscript. All authors read and approved the final manuscript.
